# A Closer Look at the *eve* Stripe 2 Enhancers of *Drosophila* and *Themira*

**DOI:** 10.1371/journal.pgen.1000276

**Published:** 2008-11-28

**Authors:** Justin Crocker, Albert Erives

**Affiliations:** Department of Biological Sciences, Dartmouth College, Hanover, New Hampshire, United States of America; Harvard Medical School, Howard Hughes Medical Institute, United States of America

## Introduction

Gene regulatory sequences have been investigated and/or proposed to be important targets of natural selection during animal evolution [1]–[14]. However, much controversy has been generated by the contention that they are not likely to be as important as functional protein-coding evolution given the low number of such examples established to date [15],[16]. However, an important obstacle in identifying such sequences is our lack of understanding of the organizational basis for such sequences. Such an understanding could allow the rapid identification and annotation of gene regulatory functions in sequenced genomes.

Gene regulatory sequences function by displaying clusters of sites for DNA sequence-specific binding factors. Such clusters are called *cis*-regulatory modules (CRMs), of which the transcriptional enhancers constitute a large and important class. The degree to which the constituent binding elements of enhancers are necessarily organized by position, orientation, and relative spacing in order to function will dictate the constraints governing enhancer evolution. Thus, the internal functional organization of enhancers is important for understanding the mode and tempo of gene regulatory evolution as well as for deciphering and annotating genomic sequences.

Arguably, no other metazoan *cis*-regulatory module has yet been as genetically and biochemically defined as the *even-skipped* (*eve*) stripe 2 enhancer module of *Drosophila melanogaster* [17]–[23]. For this reason, this module has been intensely studied from a phylogenetic perspective amongst drosophilids [24]–[27]. These phylogenetic studies of the *eve* stripe 2 enhancer have now been extended into *Themira*, a sepsid fly [28]. This latest study is used to make a central claim that a lack of sequence conservation between the *eve* stripe 2 enhancers of *Drosophila melanogaster* and *Themira putris* suggests that “complex animal regulatory sequences can tolerate nearly complete rearrangement of their transcription factor binding sites”. Thus, this study is being interpreted to reach conclusions addressing an important ongoing debate on the degree of functional organization of enhancers [29]. The results of this debate therefore impact the much larger discussion on the genetic loci of evolution [15],[16].

Both *Drosophila* and *Themira* are acalyptrate flies and last shared a common ancestor at least ∼110 Mya, and so it is suggested that this distance is long enough for the sequences to be completely scrambled in divergent organisms still sharing a similar embryonic patterning system. While the sepsid study presents an informative taxonomic collection of an evolving enhancer, this study falls short in critically testing the claim that enhancer organization is not important. Moreover, here we report that we find extensive homology in what is claimed to be an exemplar of scrambled enhancer sequences. As explained below, these ordered blocks of homology encompass well-known activator and repressor binding sites. Thus, the organization of Acalyptratae *eve* stripe 2 enhancers has not diverged enough to rule out organized assembly of higher-order enhancesome complexes at these sequences.

### Extensive Homology in the *eve* Stripe 2 Enhancers of *Drosophila* and *Themira*

**Figure 1:**
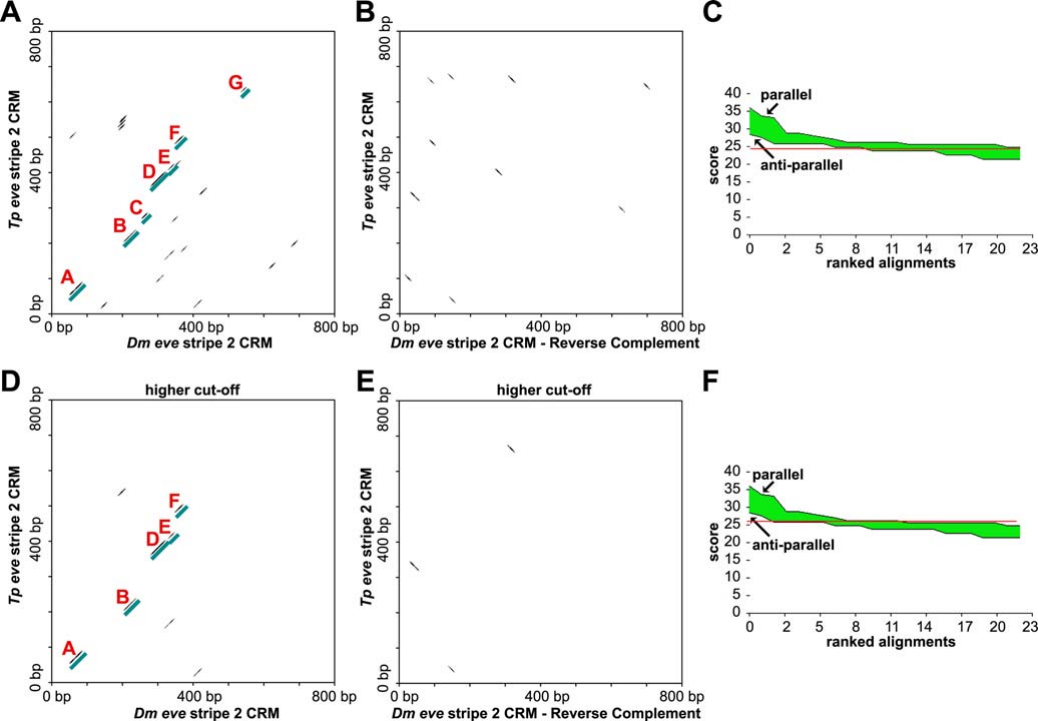
Two-dimensional dot plots of the *eve* stripe 2 enhancers of *Drosophila* and *Themira*. (A) A two-dimensional dot plot for parallel orientations of a pair of *Drosophila* and *Themira eve* stripe 2 enhancers, which have been diverging for at least ∼110 My, shows extensive blocks of conservation that are maintained in the same serial order in each species (see blocks labeled A–G in red). (B) Shown is the anti-parallel orientations of the same sequences as in (A) except that the reverse complement of one of the sequences is used in order to find additional, possibly compensatory, sites that may have changed in their orientation. Plotting the anti-parallel alignments also serves as an internal negative control. (C) A plot of the ranked alignment scores is shown for both the parallel and anti-parallel enhancer pair orientations. The score corresponds to the number of nucleotides of perfect identity within the un-gapped block of alignment. This plot shows that there are more extensive blocks of alignment in the parallel orientation than in the anti-parallel orientation. This is consistent with basic conservation of the entire enhancer. The red line indicates the threshold used for plotting points in (A) and (B). (D–F). Same as (A–C) except a higher or more stringent threshold is used. Note that a broken identity line of highly conserved blocks is easily seen in the parallel enhancer orientations (D), while most of the blocks of alignment seen in (B) disappear in the anti-parallel orientation at this stringency (E).

We first began by graphing the *Themira* and *Drosophila* stripe 2 enhancer sequences on two-dimensional sequence alignment plots (Figure 1). Such a dot plot or graphic matrix shows all regions of similarity between two sequences [30]. Such an alignment is helpful for visualizing possible insertions, deletions, rearrangements, inversions, repeats, and overall homology, without being constrained by global alignments. We also computed the same dot plot using the reverse complement of one of the sequences (Figure 1B and 1E). In addition to showing similar sequences that happen to occur in the opposite orientation, graphing the reverse complement serves as an internal negative control for conservation of serial blocks of sequence. Here, we report that when we graph the *eve* stripe 2 enhancers in parallel orientations, we see large blocks of alignment spanning ∼600 bp, almost the entire length of the enhancer (Figure 1A). These blocks are larger and more numerous compared to the number and types of alignable blocks achieved when we align them in anti-parallel orientation, i.e., when we plot against the reverse complement of one of the sequences (compare Figure 1A and 1D versus 1B and 1E, or see score distributions in 1C and 1F, respectively). We made such plots for two different thresholds that correspond to an ∼14 bp length of alignment that would encompass most binding sites (Figure 1A–1C) as well as a more extensive ∼20 bp length of alignment (Figure 1D–1F). At the more stringent level, most of the alignments in the anti-parallel direction are lost (Figure 1E). However, a clear identity line of ordered blocks of conservation is visible in the parallel alignment (Figure 1D). Thus, there exists ordered blocks of highly conserved sequence of a length consistent with multiple binding sites spanning the length of the enhancer.

**Figure 2:**
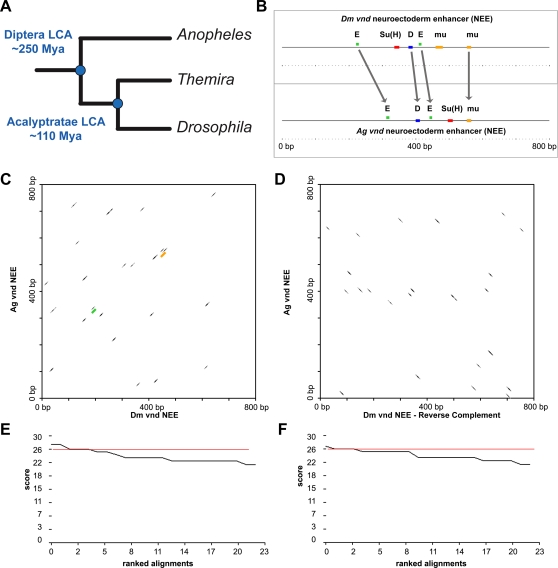
Evolutionary scrambling at the *Drosophila* and *Anopheles vnd* neuroectoderm enhancers (NEEs). (A) Shown is a phylogenetic tree of the three dipteran species discussed in the study: the sepsid fly *Themira putris*, the fruitfly *Drosophila melanogaster*, and the mosquito *Anopheles gambiae*. The amount of divergence from their latest common ancestors (LCA) is depicted in Millions of years ago (Mya). (B) The *Drosophila* and *Anopheles vnd* enhancers still share a common organization of functional binding sites as previously reported [30]. The colored boxes represent matches to the Dorsal (blue), Twist (green), mu (orange), and Su(H) (red) motifs. Two-dimensional homology plots for the *Drosophila* and *Anopheles vnd* enhancers in parallel (C) and anti-parallel (D) orientations reveal spurious blocks of alignment, as would be seen between two DNA sequences chosen randomly. Only two of the motifs shown in (B) (highlighted in green and orange) appear in the plot in (C) as indicated. The anti-parallel two-dimensional plot of these enhancers does not differ qualitatively or quantitatively from the parallel plot. (E–F) Score distributions for (C) and (D), respectively, are quite similar as well. Therefore, it is difficult to rule out organized enhancer elements without extensive sequence inspection.

The *Drosophila/Themira* study of an embryonic enhancer of the anterior posterior (A/P) axis could have been better informed by considering the *Drosophila/Anopheles* study of an embryonic enhancer of the dorsal/ventral axis (D/V) [31]. This study analyzed homologous *vnd* neuroectodermal enhancers from both *Drosophila* and the mosquito *Anopheles*, which last shared a common ancestor at least ∼250 Mya (Figure 2A). This study shows that core *cis*-elements are organized in a similar architectural plan (Figure 2B). Moreover, this conserved organization was present in *non-homologous* neuroectodermal enhancers that had evolved in parallel at other loci [31],[32]. However, the *Drosophila* and *Anopheles vnd* enhancers are so scrambled that it is difficult to find any evidence of serial sequence homology unlike the *Drosophila/Themira* pair (Figure 2C and 2D). This is consistent with the additional ∼140 My of divergence between Acalyptratae and mosquitoes on top of the ∼110 My of divergence between the *Drosophila* and *Themira* (Figure 2A).

The lesson in the mosquito example that should have informed the sepsid *eve* stripe 2 study is that the absence of extensive sequence homology is not indicative of the absence of conserved organization of binding sites. Therefore, a simple claim that an enhancer is scrambled is insufficient grounds to rule out functional organization of sites. However, in this particular case, the sepsid enhancer is actually more conserved than the *Anopheles* enhancer relative to each of their *Drosophila* orthologs (compare graphs and score in Figures 1 and 2). Below we show that these blocks of alignment in Acalyptratae sequences correspond to known transcription factor binding sites.

### Activator and Repressor Binding Sites in the Highly Ordered Blocks of Conservation

**Figure 3:**
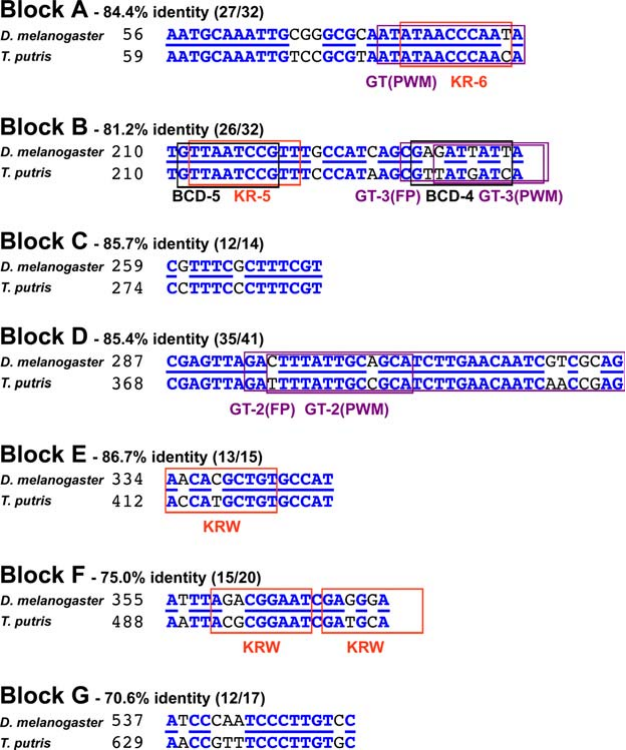
Sequence for identity blocks between *Drosophila* and *Themira eve* stripe 2 enhancers. The seven blocks of conservation shown in Figure 1, blocks (A–G), correspond to sequences encompassing well-known binding sites for Bicoid, Kruppel, and Giant in the *Drosophila eve* enhancer. The percent identity is given for each block. Abbreviations: GT, Giant; KR, Kruppel; BCD, Bicoid; KRW, weak Kruppel, i.e., low-affinity Kruppel binding; PWM, position-weighted matrix; FP, biochemical footprint.

**Figure 4:**
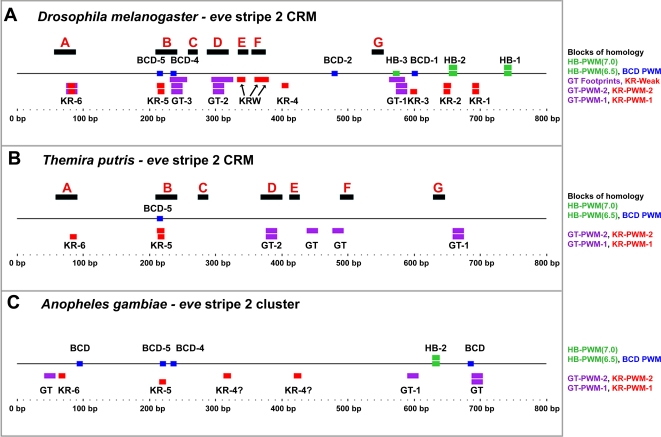
Organization of dipteran *eve* stripe 2 enhancers. The seven blocks of conservation whose serial order is conserved over a stretch of 500–600 bp across the *eve* stripe 2 enhancers of *Drosophila* and *Themira* are depicted. The blocks are shown in the order depicted in Figure 1A, blocks A–G. The colored boxes represent matches to Hunchback PWMs at two levels of stringency (lime green), a Bicoid PWM (blue), a Giant PWM (purple), and Kruppel PWMs at two levels of stringency (red). DNA binding activities for Giant and Kruppel as determined by biochemical assays are also depicted. Numbering system follows previous studies [24],[25].

There are seven large blocks of alignment between the *Drosophila/Themira eve* stripe 2 enhancers, and these span the entire length of the enhancer (Figures 1, 3, and 4). A priori, such blocks of alignment are typical of evolution at insect regulatory modules that preserve binding sites while experiencing relatively greater amounts of turnover, insertions, and deletions within intervening sequences. We began by locating in the conserved blocks of the *Drosophila/Themira eve* stripe 2 enhancers all of the well-known sites as indexed in the original biochemical and phylogenetic studies [18]–[25]. We use position-weighted matrices (PWMs) only when they accurately call the experimentally confirmed sites in *D. melanogaster* with high specificity. We note that this conservative technique may result in under-calling of *Themira* sites, including organized sites, because the position-weighted matrices were developed to *Drosophila* sequences, and because the *Themira* binding preferences may have diverged since their latest common ancestor, resulting in an artifactual phylogenetic decay of detection. Nonetheless, here we report that these seven large blocks of alignments, which are present in a conserved order or serial arrangement in both species, correspond to well-known binding sites for both activators and repressors (Figures 3 and 4).

Specifically, two high-affinity Kruppel repressor binding sites, KR-6 and KR-5, occur in conserved blocks A and B, respectively, while one and two low-affinity Kruppel binding sites (KRW sites) are present in conserved blocks E and F, respectively (Figures 3 and 4). Thus, this organized array of conserved Kruppel repressor binding sites spans ∼300 bp. Both low and high affinity sites are likely to be important in precisely reading out gradients of repressor activity [19]–[22]. Additionally, Bicoid activator binding sites BCD-5 and BCD-4 are present in conserved block B. Last, known Giant repressor binding sites are present in blocks B and D. Block D, the largest block of alignment at 41 bp, also corresponds quite well to the large biochemical footprint for *Drosophila* Giant at this site [20]. Two other conserved blocks, blocks C and G, are conserved and present in the same order in both species, but do not match any known biochemical activities. Thus, five of the seven blocks of alignment, each corresponding to a length wider than the typical binding motif, encompass well-known activator and repressor binding sites conserved in a basic order spanning the length of the enhancer for each species. This organization is of a much longer range than the conservation of adjacent binding sites noted in the study.

**Figure 5:**
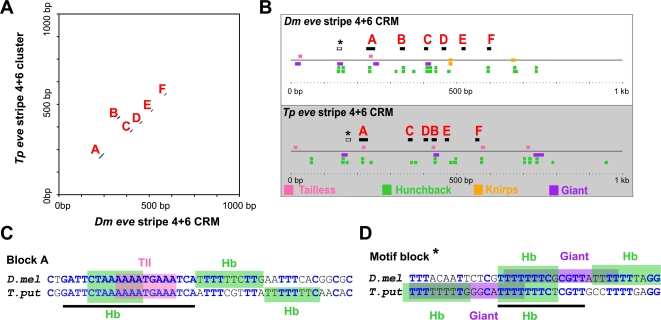
Similar amounts of organization at the *eve* stripe 4+6 enhancers of *Drosophila* and *Themira*. (A) A dot plot alignment of the *eve* stripe 4+6 enhancers of *Drosophila* and *Themira* at high stringency reveals highly conserved blocks of sequence, most of which are conserved in their basic order. (B) The blocks of alignment correspond to binding motifs for factors known to work at this enhancer: Tailless (pink), Hunchback (green), Knirps (orange), and Giant (purple). The block marked with an asterisk corresponds to sites that have been recreated in the same location, see also (D). Consequently, these sites do not appear in the parallel homology plot in (A). (C) An example of one of the blocks of conserved sequence, block A, is depicted. (D) An example of a block of sequence (asterisk) that has apparently experienced compensatory mutations that have shifted binding sites enough to obliterate homology. Despite such sequence turnover, such signatures preserve the higher-order organization of the enhancer.

Similar analyses at other *even-skipped* enhancers for A/P modulated stripes reveals a similar conserved organization of binding sites (Figure 5). For example, the *eve* stripe 4+6 enhancer contains ordered blocks corresponding to known Hunchback, Tailless, and Knirps binding sites (e.g., see Figure 5C). Additionally, there are locally rearranged blocks of sequence that destroy homology, but nonetheless preserve the presence of specific sites in the same higher-order organization (e.g., Motif block * in Figure 5D). Such sequence signatures are consistent with selection for compensatory mutations preserving binding sites in equivalent micro-neighborhoods within the enhancer [26],[32],[33]. Such a process can preserve functional organization while destroying alignment homology at specific sites.

## Conclusions

The conclusion of the sepsid study is premature because the basic premise of scrambled enhancers is doubly flawed: 1) these enhancers are not scrambled, and 2) even if they were scrambled, this would be insufficient grounds to rule out the importance of enhancer-wide functional organization of motifs as demonstrated by evolution at the dipteran *vnd* enhancer. A good test of the importance of this order of functional elements would be to rearrange these sites by mutagenesis and verify whether an “imperturbable core” is or is not present in *eve* stripe 2 enhancers. In conclusion, even though we can now easily generate panoramic views of entire genomes, we should still focus on the finer details of DNA sequence and functionally test their properties before making claims on the internal fine-structural organization of individual enhancers.
